# Synergy and competition between cancer genome sequencing and epigenome mapping projects

**DOI:** 10.1186/gm557

**Published:** 2014-05-30

**Authors:** Christoph Bock

**Affiliations:** 1 CeMM Research Center for Molecular Medicine of the Austrian Academy of Sciences, 1090 Vienna, Austria; 2 Department of Laboratory Medicine, Medical University of Vienna, 1090 Vienna, Austria; 3 Max Planck Institute for Informatics, 66123 Saarbrücken, Germany

## Abstract

**Editorial summary:**

Large-scale projects in the fields of cancer genomics and epigenomics have different aims, cultures and outcomes. The author argues that by working together a complete picture of cancer biology could be painted, and he advocates the creation of an International Cancer Epigenome Consortium.

## 

Large international consortia are currently pursuing massive sequencing of cancer genomes and comprehensive characterization of the human epigenome. Both research directions have already proven their value, uncovering exciting biology and creating promising directions for novel therapies. Here, I argue that the two fields of cancer genomics and epigenomics complement each other in promoting our understanding of cancer, in part because they operate under surprisingly different paradigms. Fostering exchange as well as healthy competition between research projects following either paradigm will contribute to a more complete picture of cancer biology and could provide us with a broader spectrum of therapeutic opportunities.

## Cancer genome sequencing: completing the census of human cancer genes

The concept of cancer as a genetic disease has been well-established since the 1970s. After the initial sequencing of the human genome by the Human Genome Project, it was an obvious next step to explore how cancer genomes are altered at the DNA sequence level. The Cancer Genome Atlas (TCGA, http://cancergenome.nih.gov) was launched in 2005/2006 with a pilot study establishing the feasibility of large-scale genome characterization in three types of cancer. Building on the success of this pilot, TCGA was extended to more than 20 different cancers in 2009. Furthermore, the International Cancer Genome Consortium (ICGC, https://icgc.org/) was founded in 2008 with the goal of coordinating cancer genome projects worldwide, comprising 25,000 samples in 50 cancer types.

The defining goal of these large-scale cancer genome sequencing projects is to establish a complete census of cancer genes [1], in much the same way as the field of genetic epidemiology is working on a comprehensive table of genetic disease risks through massive-scale association studies. The beauty of this approach lies in part in its clear and measurable goal, but also in the prospect that very large sample numbers will provide conclusive statistical evidence for disease relevance even when a detailed understanding of the biological mechanisms is lacking. Completing the census of human cancer genes is expected to contribute not only to improved risk stratification for cancer patients, but also promises to systematically identify many new targets for cancer drug development [2].

However, this reductionist focus on cataloging recurrent genetic alterations is not only a strength but also a limitation. Because most genetic alterations in cancer are rare, even an optimistic calculation suggests that it will take around 100,000 cancer genomes to finalize the cancer gene census for the most common cancer types [3], and this number is probably going to increase because detailed molecular investigations often subdivide common cancers into collections of much rarer diseases. In rare cancers, even a worldwide network for sample collection would not suffice for building a statistically sound catalog of recurrent genetic alterations. This problem is further aggravated when combinatorial effects are taken into account, and for the majority of patients that present with several rare mutations there will not be any other patient in the databases with the exact same combination of mutations.

## Epigenome mapping: charting complexity beyond the genome

Whereas cancer genome sequencing assumes that cancers are driven by relatively few and well-defined genetic alterations, epigenetic research has been more inclined to embrace complexity, stochasticity and interactions with the environment as key elements of cancer biology [4]. Epigenetic alterations are as widespread in cancer as genetic alterations, and they have been observed in every single tumor sample that has so far been studied using high-resolution epigenome mapping technology. Furthermore, DNA methylation is mitotically heritable and strongly associated with gene repression; hence, it is likely that at least some epigenetic alterations can be drivers of clonal evolution in much the same way as genetic alterations. But there is also a global dimension to epigenetic alterations that distinguishes them from the localized nature of most genetic alterations. Epigenetic profiles are highly cell-type-specific and undergo reprogramming when cells differentiate, dedifferentiate or otherwise alter their cell state. Genome-wide alterations of epigenetic marks can also be induced by exposures to environmental influences, and such induced changes may be maintained across cell divisions even after the initial stimulus has disappeared.

To create a reference framework for studying epigenetics in cancer and other diseases, an international human epigenome project has been proposed by a working group of the American Association for Cancer Research (AACR) in 2005 [5], building upon proof-of-concept studies in Europe, in the US and elsewhere. This proposal contributed to the establishment of the Roadmap Epigenomics Project in 2007/2008 (http://www.roadmapepigenomics.org) and to the formation of the International Human Epigenome Consortium (IHEC) in 2010 (http://ihec-epigenomes.org). Many national and international initiatives have joined the IHEC in its goal to establish comprehensive reference epigenomes for a total of 1,000 cell types from healthy and diseased donors. Examples include the European BLUEPRINT (http://www.blueprint-epigenome.eu), the German DEEP (http://www.deutsches-epigenom-programm.de) and the Japanese CREST-IHEC (http://crest-ihec.jp) projects.

Epigenome projects have been more open-ended than cancer genome sequencing, pursuing a broader range of goals and incorporating a larger amount of hypothesis-driven research. While the study of cancer has always been central to epigenetic research [6] and continues to be a major priority, epigenome projects have also contributed substantially to our understanding of pluripotency and cellular differentiation, and the resulting insights have helped devise improved methods for cellular reprogramming and *in vitro* differentiation. The hematopoietic system has also become a major focus of ongoing epigenome projects, and this relatively accessible and well-characterized lineage is providing important insights into the mechanisms of cellular differentiation *in vivo*.

## Perspectives for an international cancer epigenome consortium

When the TCGA and ICGC projects were conceived, epigenome mapping was in its infancy and difficult to perform on primary tumor samples. Furthermore, there was substantial skepticism in the cancer genomics community as to whether epigenetic aberrations were functionally important or maybe just downstream effects of changes in classical cancer signaling pathways. For these reasons, epigenome mapping does not play a major role in ongoing cancer genome projects, where it has largely been restricted to DNA methylation mapping of preselected genomic regions using a commercial microarray platform. However, recent developments have convincingly refuted both concerns. First, painstaking technology optimization has made it possible to establish comprehensive epigenomes - comprising DNA methylation and its oxidized variants, multiple histone modifications, chromatin accessibility and the coding and non-coding transcriptome - in limited amounts of primary patient samples; and ongoing efforts could even enable genome-wide DNA methylation analyses in single cells. Second, the important functional role of epigenetic mechanisms in cancer has been conclusively established by the identification of recurrent genetic aberrations in several dozen epigenetic regulator genes across a wide range of cancer types [7].

In light of these recent developments, the AACR working group that initiated the discussions for the formation of IHEC has proposed that the time is ripe for establishing an International Cancer Epigenome Consortium (ICEC), which could take the concepts of the IHEC forward and more specifically contribute to the development of new cancer therapies [8]. The cornerstone of such a project will be the comprehensive characterization of epigenomes in a large number of cancer samples. Furthermore, because of the complexity of cancer epigenomes and the many ways in which they could contribute to cancer, the mapping component should be complemented by: (i) functional studies dissecting cause and consequence in cancer genomics (for example, using emerging methods for epigenome editing); (ii) bioinformatic modeling of the interplay of genetic and epigenetic changes; (iii) comprehensive characterization of the mechanisms of action for existing and new epigenetic drug candidates; and (iv) the development of epigenetic biomarker candidates into relevant diagnostic assays for personalized medicine.

A cancer epigenome project designed along these lines would be highly complementary to existing efforts in cancer genome sequencing. Its focus on the complexity of cell states and their reprogramming by the cellular environment, signaling pathways and cancer drugs would provide an important counterbalance to the reductionist approach taken by cancer genome sequencing. Although there will be some overlap in the experimental assays (for example, with DNA methylation mapping), the different analysis paradigms would result in very different conclusions. Rather than cataloging single epigenetic drivers and their statistical significance, a cancer epigenome project that is inspired by the success of IHEC could focus on the concept of epigenetic cell states [9] and cellular reprogramming through individualized combination therapies [10] as novel approaches for interfering with cancer development, progression and drug resistance. In summary, the paramount importance of epigenetics in cancer has been convincingly shown over the course of the past 5 years, and it is unquestionable that an internationally coordinated and adequately funded initiative in the field of cancer epigenomics could have major impact.

## Abbreviations

AACR: American Association for Cancer Research; ICGC: International Cancer Genome Consortium; IHEC: International Human Epigenome Consortium; TCGA: The Cancer Genome Atlas.

## Competing interests

The author declares that he has no competing interests.
